# Assessment of the impact of HIV infection and anti-retroviral treatment on the cardiometabolic health of pregnant mothers and their offspring (ARTMOMSBABES)

**DOI:** 10.1186/s12872-021-02130-2

**Published:** 2021-06-30

**Authors:** Benedicta Ngwenchi Nkeh-Chungag, Godwill Azeh Engwa, Charles Businge, Mziwohlanga Mdondolo, Magdevy Pajaro Medina, Nandu Goswami

**Affiliations:** 1 Department of Biological and Environmental Sciences, Faculty of Natural Sciences, Walter Sisulu University PBX1, Mthatha, 5117 South Africa; 2 Department of Obstetrics and Gynaecology, Walter Sisulu University, Nelson Mandela Academic Hospital, Mthatha, 5117 South Africa; 3 Department of Obstetrics and Gynaecology, Mthatha Regional Hospital, Private Bag x 5014, Mthatha, 5099 South Africa; 4 Department of Peadiatrics, Mthatha Regional Hospital, Private Bag x 5014, Mthatha, 5099 South Africa; 5 Physiology Division, Otto Loewi Research Center for Vascular Biology, Immunology and Inflammation, Medical University of Graz, Neue Stiftingtalstrasse 6, 8036 Graz, Austria

**Keywords:** Cardiovascular diseases, Anti-retroviral treatment, Human immunodeficiency virus, Epigenetics, Endothelial dysfunction

## Abstract

**Background:**

The risk of cardiovascular diseases (CVDs) is becoming more prevalent in pregnant women though not much data is available for pregnant women with human immunodeficiency virus (HIV). Foetoplacental vascular endothelial dysfunction is thought to be at the origin of chronic diseases such as diabetes and obesity later on in life. Because HIV and anti-retroviral treatment (ARTs) are associated with endothelial dysfunction, children exposed in utero to these conditions may be at greater risk of developing CVDs. Despite the high prevalence of HIV in pregnant South African women, little is known about the effects of ART on the cardiovascular health of the mother and offspring. Hence, the proposed study intends to investigate how HIV/ARTs may affect the cardiovascular health of the mother and offspring at different time points during the pregnancy and up to 2 years after birth.

**Methods:**

A longitudinal case–control study in HIV positive pregnant women on ART and HIV negative pregnant women will be conducted. All pregnant women will be assessed for cardio-metabolic risk factors and markers (lipids, anthropometric and glycaemic indies, oxidative stress), hemodynamic status (blood pressure parameters) and vascular function (arterial compliance, retinal microvasculature, uterine artery mean pulsatility index). Child health will be monitored in utero and postnatally via routine foetal health screening, placental integrity, anthropometry, blood pressure parameters, markers of oxidative stress and endothelial function in cord blood and cardiovascular epigenetic markers in urine.

**Discussion:**

There is a paucity of studies in South Africa and sub-Sahara Africa as a whole that utilised a longitudinal study model to assess the effects of ARTs on vascular endothelial changes in pregnant women living with HIV and the cardiometabolic health of their offspring. This study will therefore help to monitor changes in cardiometabolic risk during pregnancy and in children exposed in utero to HIV-infection and ART use. Findings from this study will provide useful information for developing guidelines on the use of ARTs in pregnancy and management of cardiometabolic health of the offspring of HIV positive mothers.

**Supplementary Information:**

The online version contains supplementary material available at 10.1186/s12872-021-02130-2.

## Background

According to the World Health Organisation (WHO), approximately 17.9 million deaths were recorded due to cardiovascular diseases (CVDs) globally in 2016 which translates to about 31% of all-cause mortality [1]. It should be noted that 75% of these deaths occurred in low and middle income countries [1]. Risk factors for the development of CVDs include obesity, hypertension, dyslipidaemia, endothelial dysfunction, inflammation, microalbuminuria, insulin resistance, oxidative stress, etc. [2]. The prevalence of some of these risk factors is very high in the general South African population: obesity (20–25%), hypertension (10.2%), dyslipidemia (24%) and insulin resistance (10–20.7%) [3] thus explaining why CVDs may contribute significantly to death rates in South Africa [4]. Vascular abnormalities such as arteriolar-venular nicking and increased carotid intima–media thickness, and focal and generalized arteriolar narrowing in adults have been associated with CVDs [5, 6]. Changes in arterial structure generally take a long time to be discernible in the larger vessels. Detectable changes however are more readily noticeable in the smaller vessels thus presenting an opportunity for early assessments. These microcircular changes can be non-invasively assessed and monitored in the retina using fundoscopy [7–10]. Whereas widening of retinal venules is linked to endothelial dysfunction and inflammation, narrowing of retinal arterioles is linked to increased blood pressure and endothelial dysfunction. Both of these changes in the retinal microvascular are indicative of endothelial dysfunction, a major cardiovascular risk factor.

Recent reports show that people living with HIV (PLWH) have an abundance of traditional CVD risk factors and may therefore be at increased risk of CVDs [11, 12]. Indeed, a prospective cohort study for a 6-year period with over 80,000 HIV-infected persons showed that HIV-infected adults with hypertension had a twofold higher risk of developing acute myocardial infarction than uninfected adults with hypertension [13]. Also, studies have shown obesity and dyslipidaemia to be prevalent in HIV patients [14]. In South Africa, the prevalence of CVD risk factors in HIV continues to rise creating a major public health concern for the country [15, 16]. HIV/AIDS seems to be on a collision course with a rapidly increasing burden of cardiovascular risk factors and disease in this population [17–19]. Indeed it is projected that by 2030, about 84% of HIV-infected patients will be affected by at least one non-communicable disease with over one-third of HIV patients having three or more non-communicable diseases [20].

While PLWH now live longer, thanks to the availability of anti-retroviral treatments (ARTs) though there are concerns that some ARTs may be associated with dyslipidaemia, hypertension and dysglycaemia [21]. Studies have shown that ARTs are associated with dysregulation of plasma lipid levels and metabolism [22–24]. Also, ARTs may affect glucose homeostasis and promote insulin resistance [21]. Furthermore, HIV-infected adults on ART have a higher prevalence of hypertension when compared with HIV-uninfected individuals [25]. These findings seem to suggest that ARTs may improve the life expectancy of PLHW but also make them more vulnerable to developing CVDs on the long run. Also, ART may cause direct endothelial injury and promote endothelial dysfunction [26], which is an important risk factor for CVDs. Although ART and HIV have been associated with CVDs, such findings are yet to be established in HIV pregnant women [27, 28].

Globally, over 1.4 million pregnant women are living with HIV [29]. Among this population, about 960,000 HIV-infected pregnant women reside in Eastern and Southern Africa. In South Africa, 30.2% of all pregnant women who attended public health-care facilities in 2012 were infected with HIV [30]. The risk of CVD is becoming more prevalent in pregnant women [31] though not much attention has been paid to this population. Gestational diabetes mellitus (GDM) is associated with macro and microvascular endothelial dysfunction of the placenta mainly triggered by hyperinsulinemia, hyperglycaemia, and changes in nucleoside extracellular concentration [32].

Endothelial dysfunction may alter the intrauterine environment of the foetus. Foeto-placental vascular endothelial dysfunction may cause epigenetic alteration in the intrauterine environment of the foetus. This may be the origin of chronic diseases such as diabetes and obesity in childhood predisposing children to CVDs which may track into adulthood [33]. According to the concept of the Developmental Origins of Health and Disease, the maternal environment/gestational milieu may have a significant impact on the health of the offspring in adulthood and may be responsible for programming children for CVDs later on in life [34]. Indeed, poor placental perfusion as in pre-eclampsia has been associated with preprograming for obesity, hypertension and diabetes in adult life [35]. Animal studies have shown that administration of combination ART during pregnancy was associated with poor placental insertion and development resulting in growth restricted offspring [36]. Because HIV infection and ARTs are associated with increased cardiovascular risk factors including endothelial dysfunction, we hypothesize that HIV-infection/ARTs will promote endothelial dysfunction which will engender poor placental perfusion resulting in offspring being pre-programed for increased cardiovascular risk later on in life.

Though PLWH now enjoy a longer lifespan following the introduction and ready availability of ARTs, the long-term safety of ART use among pregnant women is not known [37], much less its impact on the foetus, neonates and children born after in utero exposure to these drugs. There is currently no information on epigenetic changes if  in children exposed to HIV infection/ART environment or how these changes may relate to preprograming for increased susceptibility to cardiometabolic conditions later on in life. Furthermore, there is not yet a scientific explanation for the high prevalence of CVDs in young Africans and their high vulnerability to target organ damage at earlier age compared to other races [38]. South Africa has the highest prevalence (13.0%) of HIV infection globally and at least 68% of all patients diagnosed with HIV are on ARTs [39] thus making South Africa the ideal setting for determining the cardiometabolic effects of HIV/ART use on mother and offspring.

## Methods/design

### Aim and objectives

The aim of this study is to investigate whether HIV-infection and ART are associated with risk of cardiometabolic diseases in pregnant women of African ancestry and their offspring after birth. This study aims to:

1. Assess cardiometabolic disease risk in HIV positive pregnant women on ART and HIV negative pregnant women.
2. Determine foetal health in the maternal HIV/ART environment during the three trimesters of gestation.
3. Assess cardiometabolic health of mothers exposed to HIV/ART at various time points after delivery.
4. Assess cardiometabolic risk in offspring of HIV positive mothers who were on ART during pregnancy at birth at predetermined time points after birth.

### Ethical approval

Ethical consent and permission to carry out the research project was obtained from the Faculty of Health Sciences Research and Ethics Committee (HRSEC No: 017/2020) at Walter Sisulu University (WSU). The study was approved by the Eastern Cape Provincial Department of Health, South Africa and then registered with the NIH (Protocol ClinicalTrials.gov Identifier: NCT04763668; https://clinicaltrials.gov/ct2/show/NCT04763668). The purpose of the study will be explained to women attending the antenatal clinic in the Mthatha Regional and Nelson Mandela Academic Hospitals, Mthatha, Eastern Cape Province. Pregnant women who will be willing to participate will be required to sign informed consent forms to participate in the study and to allow their children to participate in the study from birth.

### Selection criteria

Pregnant women of African ancestry with singleton uncomplicated 11–14 week old pregnancies, who are either HIV positive for the case group or HIV negative for the control group will be recruited for the study. Pregnant women with type 2 diabetes, gestational diabetes, renal and CVDs or any critical health condition will be excluded from the study. Furthermore, data will not be collected from eligible pregnant women who have eaten, smoked or done physical exercise 6 h prior to data collection.

### Study design and experimental plan

A prospective case–control design will be employed for this study. This will include HIV positive pregnant women on ART and HIV negative pregnant women who will serve as control. HIV positive pregnant women below 35 years of age with singleton pregnancy on ART attending antenatal clinic at  Mthatha Regional  Hospital and Nelson Mandela Academic Hospital, Mthatha will be recruited for the study and followed up during pregnancy for the presence of cardiovascular risk factors. In addition, foetal health and cardiometabolic health of the babies born to recruited women will be assessed after birth and up to 24 months after birth.

Recruited, pregnant women with known HIV positive status and who have been on ART for over 4 months will be counselled and screened for HIV to confirm their status. Thereafter, pregnant women will be assessed during antenatal visits in the first trimester (11–14 weeks), second trimester (24–26 weeks) and third trimester (35–37 weeks) for cardiometabolic risk factors including obesity, hypertension, albuminuria, insulin resistance, dyslipidaemia, oxidative stress and endothelial dysfunction. The viral load and CD4 count will be determined in the mothers at baseline and third trimester of pregnancy. A structured interviewer administered questionnaire will be used to collect data on their baseline characteristics including demographic, obstetric and family history of CVDs while anthropometric and blood pressure measurements will be performed. Placental morphometry will be assessed by ultrasound while vascular health will be assessed via pulse wave velocity (Vicorder, SMT GmbH, Germany) and endothelial function markers in blood. Microvasculature assessment will be done via retinal imaging using a retinal camera (Optomed, Finland) as previously reported [40]. Blood samples will be collected for the assessment of endothelial function markers [41], oxidative stress, lipid profile and insulin resistance markers [42] while urine will be collected for assessment of renal function. Mothers will have one post delivery follow-up for CVD risk assessment.

Foetal health will be assessed ultrasonically in utero during the three trimester of pregnancy. Fetal health measurements will include biparietal diameter, head circumference, abdominal circumference, thoracic circumference, crown-rump length, crown-rump length and femur length. After delivery, In addition, at three, six, twelve and twenty-four months after birth, cardiometabolic risk factors will be assessed by determining BMI, blood pressure, albuminuria, oxidative stress, endothelial function as well as epigenetics markers in urine samples.

**Fig. 1:**
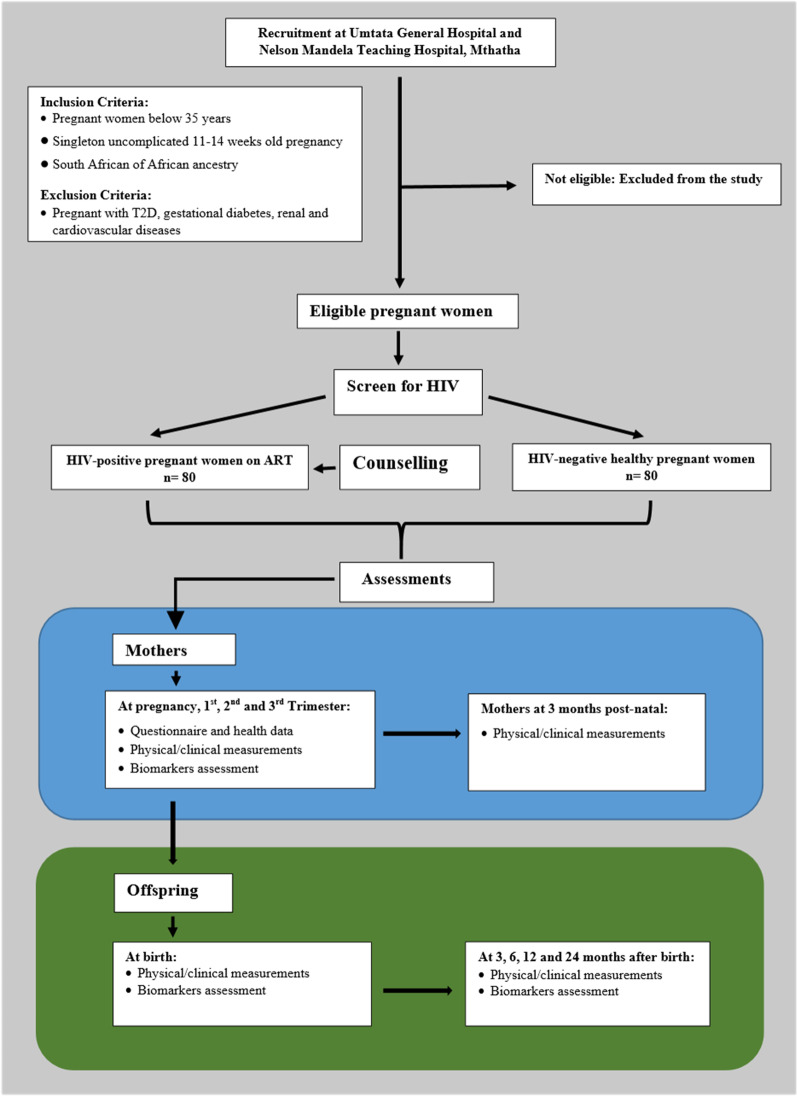
Flow diagram of study design. HIV: Human immunodeficiency virus; T2D: Type 2 diabetes

**Fig. 2:**
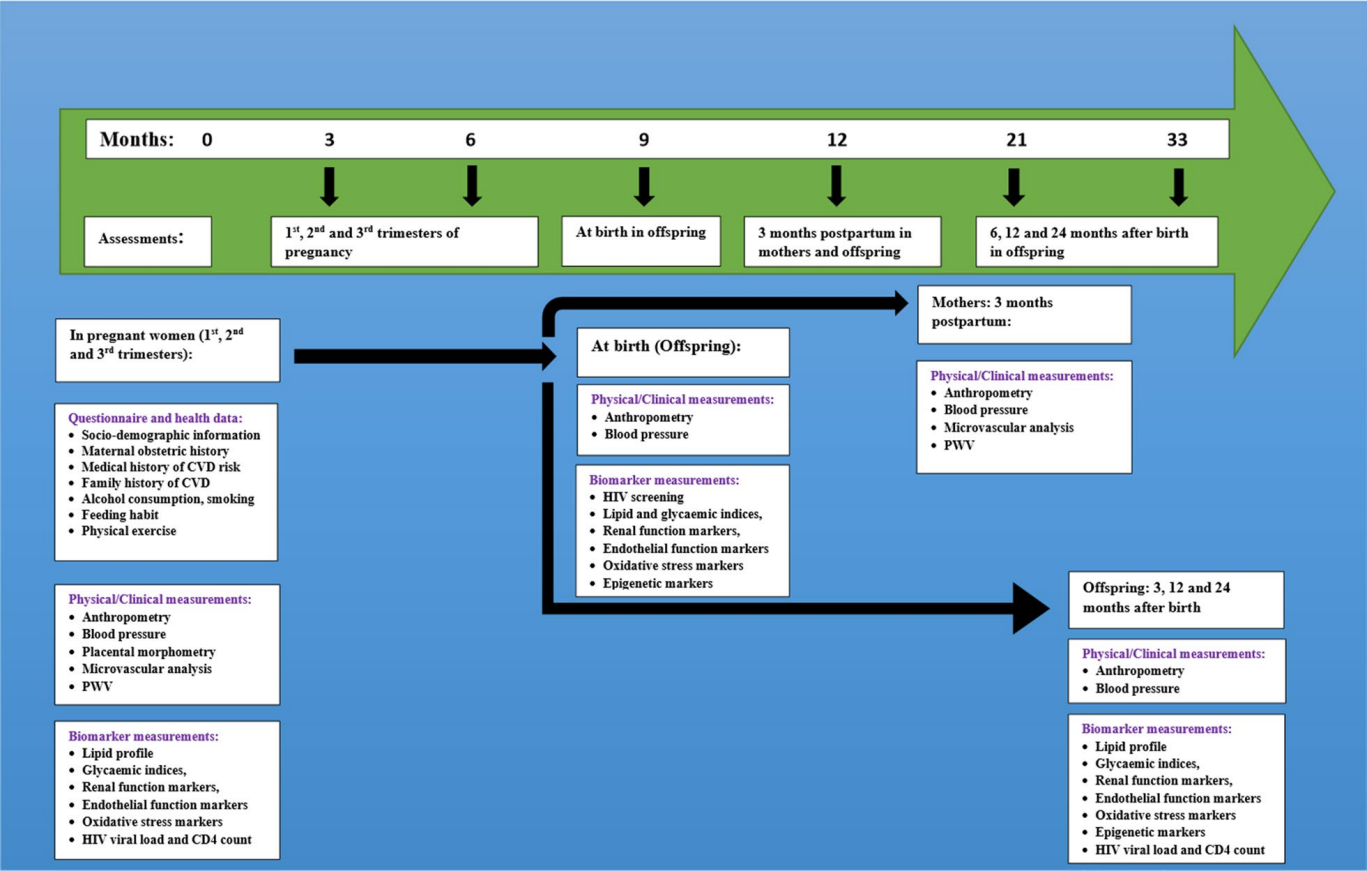
Study timeline and parameters. CVD: Cardiovascular disease; PWV: Pulse wave velocity; HIV: Human immunodeficiency virus

All pregnant participants will be required to fast overnight and will be asked to restrain from drinking coffee, smoking and doing exercise for 4–6 h before assessments. The study design along with the inclusion and exclusion criteria, and the series of assessments with the timeline are summarised in Figs. 1 and 2 respectively.

### Data collection

A structured interviewer administered questionnaire will be used for the collection of participant’s baseline characteristics and cardiometabolic risk factors (Additional file 1 - Cardiometabolic risk questionnaire). The questionnaire will contain socio-demographic information including personal data (age, sex, location and ethnicity), socio-economic status (profession, economic status, living standard). It will also contain information on maternal obstetric history, cardiometabolic health history, family history, alcohol consumption, smoking, feeding habit and physical exercise. Data will be collected using a questionnaire only at the beginning of the study.

### Physical/clinical measurements

Participants will be screened for cardiovascular risk factors by anthropometric and blood pressure measurements. Anthropometric measurements will be done according to international standards [43]. Waist and hip circumferences will be measured using an anthropometric tape and the waist to hip ratio (WHR) will be calculated. Height will be measured using a wall-mounted Harpenden stadiometer to the nearest 0.1 cm while weight will be measured using a wireless weight scale (Tanita body composition scale). The Tanita device will be used to calculate the body mass index (BMI) using the formula weight/(height (m) × height (m)). Office blood pressure including the brachial systolic and diastolic blood pressure and heart rate will be measured as described by Muntner and colleagues [44] in triplicates at three-minutes intervals after 10 min of rest in the seated position with an Omron M HEM-907XL Professional Blood Pressure Monitor (Omron Healthcare, Kyoto, Japan). This will be followed by 24 h ambulatory blood pressure measurement for determination of day time/night time blood pressure and dipping phenotype (A&D Instruments, Japan). Placental morphometry, architecture and vascularization which includes the uterine arteries, umbilical artery and middle cerebral artery, and uterine artery mean pulsality index will be determined by ultrasound.

### Clinical and laboratory investigations

Blood and early morning mid-stream urine samples will be obtained by the research nurse from each participant. Collected blood samples will be used for determining glucose, glycated haemoglobin (HbA1c), lipids (total cholesterol, LDL-cholesterol, HDL-cholesterol and triglycerides), creatinine and glomerular filtration rate (GFR), HIV viral load, CD4 count, insulin, oxidative stress markers [lipid peroxidation, total antioxidant capacity and 8-hydoxyl-2-deoxyguanine (8-OHdG)], endothelial function markers [endocan, assymetric dimethyl arginine (ADMA) and nitric oxide (NO)] and epigenetic markers (urine 5-methyl-2-deoxycytidine and trimethyl histone H3K9). Urinary albumin and creatinine concentrations will be determined in urine samples using approved standard analytical protocols. The homeostatic model assessment of insulin resistance (HOMA-IR) formula will be used to calculate insulin resistance from fasting glucose and insulin.

### Assessment of vascular functions

#### Pulse wave velocity (PWV)

PWV will be measured using the Vicorder device (SMT medical GmbH & Co. KG, Germany) as reported by Cauwenberghs and colleagues [45]. Prior to measurements, participants will lie in a supine position for 10 min after which a standard 10 cm pressure cuff will be placed on the upper right arm and blood pressure measurement taken. Thereafter, the standard 10 cm pressure cuff will be placed on the upper right thigh as high as possible towards the crotch. The centre between the base of the neck and chin will be palpated to identify the right common carotid artery pulse after which a neck band with an attached neck pressure cuff will be placed around the neck, positioning the cuff bladder exactly over the carotid artery pulse. These cuffs will be secured tightly enough to ensure a good coupling of both cuffs to the femoral and carotid arteries without discomfort to the participant. The distance between the two cuffs will be measured using a measuring tape and entered against the participants information on the Vicorder software which include height, weight and blood pressure (SBP and DBP). The vicorder device will be turned on to inflat both cuffs to about 60 mm Hg and waveforms will be recorded simultaneously over about 10 consecutive heartbeats to estimate transit time. The pulse wave velocity (m/s) will be calculated as the path length between carotid and femoral arteries/the transit time.

#### Retinal vessel analyses

Retinal vessel imaging will be captured with a non-mydriatic digital retinal camera (Optomed, Finland). A trained grader, masked to participant’s characteristics will perform the vessel measurements on the optic disc–centred image of both the right and left eye. After measurements, the retinal vessel dimensions, microvascular state and vascular tree will be analysed with semi-automated IFLEXIS software (VITO, Belgium) [46] by calculating the vessels widths and pattern features including the tortuosity, fractal analysis and lacunarity. The average diameter of venules and arterioles of the eye will be summarised as central retinal venular equivalent (CRVE) and central arteriolar equivalent (CRAE) respectively [47]. An overview of the different retinal features that will be calculated as reported in a previous study [48].

### Data management and integrity

This study will make use of the REDCap (Research Electronic Data Capture) system to capture all data elements. REDCap is a free web-based, secured and user-friendly electronic database software to support data capture and storage for research studies. It can easily be customized to suit the collection, storage and tracking of information and data from specific research studies [49]. The REDCap system will be managed by a dedicated Data Manager that will ensure the correct handling of the database. This will entail creating unique participant’s identifier number linked to their data with no association to their personal details thereby keeping participant’s data confidential.

### Proposed analysis

Data analysis which includes sample size determination and statistical analysis will be done by the project’s statistician.

#### Sample size calculation

The number of participants required to show statistical significance is based on previously published studies in which vascular function was assessed in HIV patients [50]. An error probability (α) of 0.05, and power (1 − β) of 0.80 and an average effect size (d) of 0.5 were used to calculate the sample size (n = 64 persons). Assuming a 25% drop out rate, we will use N = (64 + (64*25)/100) = 80 mothers/group to ensure that we will have enough sample size/group to show statistical significance. Total number of participating mothers will therefore be 160 (80 cases and 80 controls) and their offspring-160.

#### Statistical analysis

Data analyses will be carried out using IBM statistical package for social sciences (SPSS) Version 23.0 (IBM Corp.). Armonk, NY, USA) and STATA. Data will be checked for normality using Shapiro-Wilks test and log-transformed and/or corrected for outliers when necessary. Descriptive statistics are presented as mean ± standard deviation (SD). Mean differences between groups (HIV positive pregnant women and HIV negative pregnant women) will be done using independent sample t-test. Analysis of variances (ANOVA) will be used to compare continuous variables between three or more groups. Multivariate analysis using multiple analysis of variance (MANOVA) will be used to compare continues variables for two or more independent variables (ART, HIV etc.). Chi-square test will be used to associate cardiovascular risk factors at baseline and follow-up for both mother and child. Pearson correlation will be used to assess relationship between risk factors of CVD. Linear and binomial logistic regressions will be employed to assess the risk of CVDs due to HIV and ART. A confidence interval of 95% will be taken and α = 0.05 will be considered for level of significance.

## Discussion

This ARTMOMSBABES study intends to investigate whether HIV-infected pregnant women on ART have a higher risk for cardiometabolic conditions and whether their offspring are borne with an increased risk for similar conditions. We are not aware of any study in sub-Saharan Africa that has utilised a longitudinal study model to assess the in utero effect of HIV/ART on cardiometabolic risk factors including vascular endothelial changes in pregnant women living with HIV and a follow up of their offspring. Existing studies have shown that the use of ART is associated with cardiovascular risk in people living with HIV [51–53] but little is known about the effect of HIV-infection and ART use in pregnant women and how these may affect the offspring exposed to both the in utero. The repeated measurements in the first, second and third trimesters of pregnancy will help to monitor changes if any in the progression of cardiometabolic risk as a result of HIV-infection and ART use in pregnancy. That is, whether there exist placental vascular changes due to ART. More so, follow-up of offspring after birth will be able to establish whether in utero expose to ART has long-term effects on the cardiometabolic health of children born of HIV-infected mothers on ART.

Findings from this study will provide information that may be helpful to develop guidelines on the use of ART and management of cardiovascular health in HIV-infected pregnant women in local health care settings. It will provide information on the cardiometabolic health of children born to HIV-infected pregnant women on ART for improved management and development of prevention policies. It will also contribute to the current global knowledge on HIV and CVD in pregnant women and children. The findings of this study may also provide an explanation and understanding of the high prevalence and early occurrence of cardiovascular risk in children especially in the South African context, as current evidence has reported increased prevalence of obesity, hypertension and other CVD risk factors in children [54–57]. These findings may also contribute to the establishment of cardiovascular disease and vascular endothelial endpoints in pregnancy which may be specific to the South African and African context but may also be useful to predict future cardiovascular events in children.

The strength of this study relies on the fact that pregnant women (and the foetus) are followed up during pregnancy and thereafter their offspring followed up to investigate the possible epigenetics effect of HIV/ART with regards to cardiovascular risk between mother and offspring. Moreover, the fact that this study combines a wide array of techniques from anthropometric, to non-invasive imaging and ultrasound techniques coupled with measurements of a series of biological markers of cardiometabolic risk factors, vascular function and epigenetics provides a more holistic assessment of cardiovascular risk to attain the desired objectives.

Despite the above strengths of this study, it may be limiting in that: It will be conducted in Mthatha in the Eastern Cape Province of South Africa, a location which is highly dominated by South Africans of African Ancestry. Therefore, the study will mainly constitute South African pregnant women who are of African ancestry. Other ethnic groups within the South African population will not be considered. However, the ethnicity of the children born of these mothers may not be restricted because of the background of their fathers. This study intends to follow-up the children for a period of 24 months which may be too short to sufficiently assess the progression of vascular changes and cardiovascular risk. However, the follow-up period was chosen with respect to the funding period. Presently, the project team is building capacity to attract more funds which can further extend the follow-up period beyond the study duration. A limitation maybe also be that the risk of metabolic syndrome in the offspring may be over-estimated or underestimated when using cut-offs for cardio-metabolic risk from publications from Western world. This will be minimized by ensuring that ethnicity and sex are taken into account when interpreting the results [58–60].

The ARTMOMSBABES study is an important study in the South African context as there is evidence of increasing prevalence of cardiovascular risk in children without a clear understanding of the underlining cause. This study may be able to address this concern by first assessing cardiometabolic risk and vascular changes in HIV-infected pregnant women on ART and then follow-up their offspring after birth for possible cardiovascular risk as a result of in utero exposure. The finding may contribute to develop guidelines in the management of ARTs used in pregnant women and cardiovascular risk in children. The biomarkers analysed will be helpful to understand the molecular determinants of vascular endothelial changes in pregnancy that may promote the development of cardiovascular risk in children. More so, the findings of this study may start the process of unravelling the mechanism behind the increased prevalence of cardiovascular risk factors observed in children of African ancestry.

## Supplementary Information

**Additional file 1.** Cardiometabolic risk questionnaire.

## Data Availability

We do not wish to share the data that will arise from this study as the participants data are kept confidential in accordance with the South African National Data Protection guidelines for reporting.

## Abbreviations

8-OHdG: 8-Hydoxyl-2-deoxyguanine
ADMA: Assymetric dimethyl arginine
ANOVA: Analysis of variances
ARTs: Anti-retroviral treatments
BMI: Body mass index
CRAE: Central arteriolar equivalent
CRVE: Central retinal venular equivalent
CVDs: Cardiovascular diseases
GFR: Glomerular filtration rate
HbA1c: Glycated haemoglobin
HDL-c: High density cholesterol
HIV: Human immunodeficiency virus
HOMA-IR: Homeostatic model assessment of insulin resistance
HRSEC: Health Sciences Research and Ethics Committee
LDL-c: Low density-cholesterol
MANOVA: Multiple analysis of variance
NO: Nitric oxide
NRF: National Research Foundation
PLWH: People living with HIV
PWV: Pulse wave velocity
REDCap: Research electronic data capture
SPSS: Statistical package for social sciences
WHO: World Health Organisation
WHR: Waist to hip ratio
WSU: Walter Sisulu University
