# Supplementary material for: Assessment of the impact of HIV infection and anti-retroviral treatment on the cardiometabolic health of pregnant mothers and their offspring (ARTMOMSBABES)
Source: BMC Cardiovasc Disord. 2021 Jun 30;21:322. doi: 10.1186/s12872-021-02130-2 (PMC8247111; doi:10.1186/s12872-021-02130-2)
Supplement: Supplementary file 1 — Additional file 1. Cardiometabolic risk questionnaire. [file 12872_2021_2130_MOESM1_ESM.pdf]

Assessment of the Impact of HIV Infection and Anti-Retroviral Treatment on the Cardiometabolic Health of Pregnant Mothers and their Offspring (ARTMOMSBABES)

Date: ______________________________

DEMOGRAPHICS

Patient ID:	________________________________
First name	________________________________
Surname	________________________________
Age	________________________________
Sex	________________________________
Ethnicity	________________________________
House/Flat Number	________________________________
Dwelling type	○ House<br>○ Flat or Complex<br>○ Informal housing
Building/Complex Name	________________________________
Street name	________________________________
Suburb	________________________________
For how many years have you lived at current address?	________________________________<br>((months as decimal e.g. 6 months = 0.6))
Telephone number	________________________________

SOCIO-ECONOMIC INFORMATION

**Education**<br>What level of education have you completed?	○ None<br>○ Primary school<br>○ High school<br>○ ABET (Adult Basic Education Training)<br>○ College/University/Other tertiary institution
**Employment**<br>Which of the following applies to your current employment situation?	○ Unemployed<br>○ Employed (full time)<br>○ Employed (part time)<br>○ Self-employed
If unemployed, which of the following applies to you?	○ Looking for work<br>○ Discourage job seeker - not looking for work<br>○ Student<br>○ Homemaker<br>○ Illness/disability prevent me to work<br>○ Too old to work<br>○ Other
If other, specify?	________________________________
**Income**<br>Do you or someone in your household receive a Government Social Grant?	○ Yes

	○ No
What is the total of your household income per month?	○ less than R1,000<br>○ R1,000 - R4,999<br>○ R5,000 - R9,999<br>○ R10,000 - R20,000<br>○ more than R20,000

PREGNANCY

Are you pregnant?	○ Yes<br>○ No
If pregnant, what type of pregnancy?	○ Singleton<br>○ Twin
If pregnant, when was your last normal menstrual period?	___________________________________<br>((weeks))
What trimester is your pregnancy?	1st (1-11) weeks/1-3 months)<br>2nd (12-23 weeks/3-6 months)<br>3rd (above 24 weeks/ above 6 months)

HIV

Are you HIV positive?	○ Yes<br>○ No
If positive, are you on ART?	○ Yes<br>○ No
Which line of ART are you on?	○ 1st<br>○ 2nd
What is the name of the ART?	___________________________________
For how long have you been on ART?	___________________________________<br>((weeks))
Date you were diagnosed with HIV?	___________________________________
If date unknown, how long ago approximately were you In last year diagnosed with HIV	2 - 5 years ago<br>>5 years ago<br>>15 years ago

MATERNAL OBSTETRIC HISTORY

Is this your first pregnancy?	○ Yes<br>○ No
If No, how many times have you been pregnant?	○ Yes<br>○ No
How many children do you have?	○ Yes<br>○ No
Have any of your pregnancies ended in stillborn?	○ Yes<br>○ No
Were any of your pregnancies complicated by premature contractions or Premature delivery?	○ Yes<br>○ No
Have any of your deliveries required forceps or vacuum extraction?	○ Yes

	○ No
Have any of your deliveries required Caesarean section?	○ Yes<br>○ No

MEDICAL HISTORY

PAST

Have you had a heart attack in the past?
- ○ Yes
- ○ No

Have you had a stroke in the past?
- ○ Yes
- ○ No

Do you have type 2 diabetes?
- ○ Yes
- ○ No

Did you have gestational diabetes with previous pregnancies?
- ○ Yes
- ○ No

Do you have kidney disease?
- ○ Yes
- ○ No

Do you have any heart or cardiovascular disease?
- ○ Yes
- ○ No

PRESENT

Do you have high blood pressure (hypertension)?
- ○ Yes
- ○ No
- ○ Unknown

If yes, what year were you first diagnosed? ____________________

Do you have a heart disease?
- ○ Yes
- ○ No
- ○ Unknown

If yes, what year were you diagnosed? ____________________

Do you have high cholesterol?
- ○ Yes
- ○ No
- ○ Unknown

If yes, what year were you diagnosed? ____________________

Do you have any other long lasting health problems? (For example: kidney stones, arthritis, asthma, bilharzia, malaria)
- ○ Yes
- ○ No

If yes, please specify what the health problem is and what year you were diagnosed? ____________________

Do you currently have pulmonary Tuberculosis (TB)?
- ○ Yes
- ○ No

If yes, are you on treatment?
- ○ Yes
- ○ No

When did you start treatment? ____________________

(If the patient cannot recall date, enter year (yyyy))

DIABETES

Do you have Diabetes	o Yes<br>o No
What type of Diabetes do you have?	o Type I Diabetes (also known as Juvenile Onset or Insulin Dependent Diabetes)<br>o Type II Diabetes (also known as Non-insulin Dependent Diabetes)<br>o Don't know
How long ago were you told you have diabetes?	< 12 months<br>1-5 years<br>6-15 years<br>15+ years
Which of the following do you use to manage your diabetes?	Diet<br>Pills<br>Injection<br>Nothing<br>Other<br>((tick all that apply))
If other, please specify.	___________________________________

FAMILY HISTORY

STROKE AND HEART DISEASE

	Don't know	No	Yes, under the age of 60	Yes, over the age of 60	Yes, but I don't know the age
Has your mother had any heart disease?					
Has your father had any heart disease?					
Has your mother ever had a stroke?					
Has your father ever had a stroke?					
Does any of your sibling have a heart disease?					

DIABETES

	Don't know	No	Yes, Type I	Yes, Type II	Yes, but I don't know type
Does your mother have Diabetes?					
Does your father have Diabetes?					
Does any of your sibling have Diabetes?					

HIGH BLOOD PRESSURE AND CHOLESTEROL

	No	Yes	Don't know
Has your mother had high blood pressure?			
Has your father had high blood pressure?			
Has your mother had high cholesterol?			
Has your father had high cholesterol?			
Does any of your sibling have hypertension?			

LIFESTYLE

Are you a smoker
- o Yes currently
- o In the past
- o Never smoked

What type of cigarette do/did you smoke?
- o Snuff
- o Tobacco
- o Dagga

On average, how many cigarettes do you smoke on the days that you smoke?
- o More than 20 daily
- o Less than 20 daily

If you have stopped, how long has it been since you last smoked (months)? ___________________________________

ALCOHOL

Have you consumed an alcoholic drink within the past 12 months?
- o Yes
- o No

How often do you typically drink?
- o Daily
- o 8 or more days a month
- o Less than 8 days a month

At what age did you start drinking regularly (at least once a week)? ___________________________________
((answer in years))

WHAT DO YOU DRINK?

	o Yes<br>o No
Beer	
Spirits (brandy, vodka, cane etc.)	
Red Wine	
White Wine	
Other	
When you drink beer, how many standard units do you typically have on a single occasion? (see reference card).	___________________________________

When you drink spirits, how many standard units do you typically have on a single occasion? (see reference card).	______________________________
When you drink red wine, how many standard units do<br>you typically have on a single occasion? (see reference card).	______________________________
When you drink white wine, how many standard units do you typically have on a single occasion? (see reference card).	_<br>______________________________
When you drink other, how many standard units do you typically have on a single occasion? (see reference card) and describe.	______________________________

Alcohols standard unit reference card

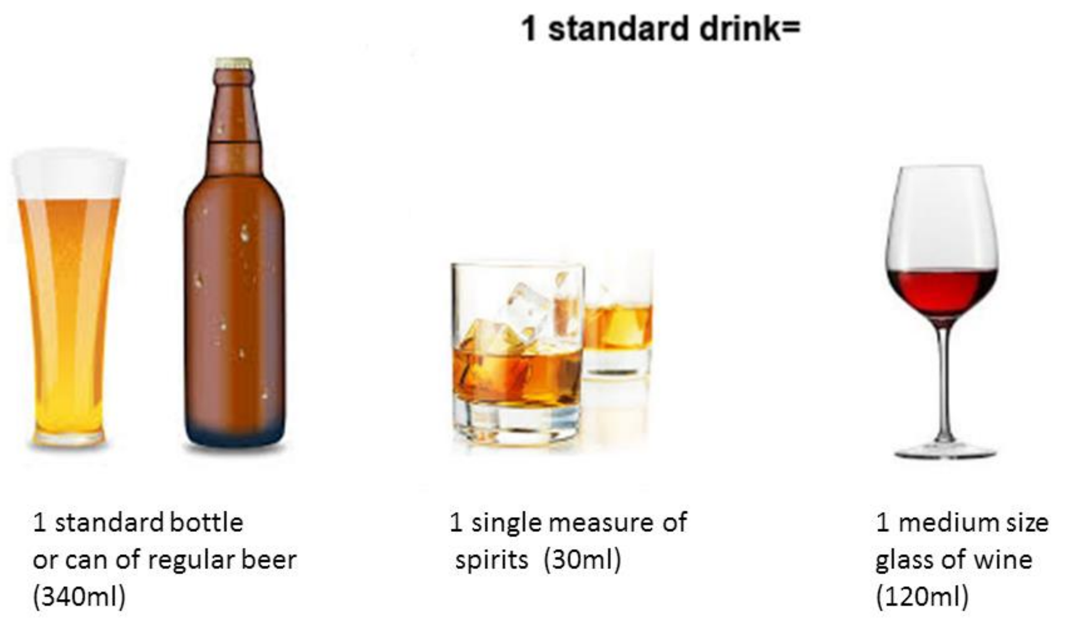

EATING HABITS

How many meals do you have on a typical day?	○ One<br>○ Two<br>○ Three<br>○ More than three
Do these meals include fruit and/or vegetables?	○ Yes<br>○ No

EXERCISE

Are you physically active?	○ Yes<br>○ No
If yes, how many times do you exercise in a week?	○ Once<br>○ Twice<br>○ Three times

	○ More than three times
How would you describe the type of exercise you do?	○ Mild<br>○ Moderate<br>○ Intensive
